# Deuterium Solid State NMR Studies of Intact Bacteria Treated With Antimicrobial Peptides

**DOI:** 10.3389/fmedt.2020.621572

**Published:** 2021-01-11

**Authors:** Valerie Booth

**Affiliations:** Department of Biochemistry and Department of Physics and Physical Oceanograpy, Memorial University of Newfoundland, St. John's, NL, Canada

**Keywords:** HDP, AMP, intact bacteria, whole cell NMR, solid state NMR, magic angle spinning (MAS), lipid membrane, bilayer

## Abstract

Solid state NMR has been tremendously useful in characterizing the structure and dynamics of model membranes composed of simple lipid mixtures. Model lipid studies employing solid state NMR have included important work revealing how membrane bilayer structure and dynamics are affected by molecules such as antimicrobial peptides (AMPs). However, solid state NMR need not be applied only to model membranes, but can also be used with living, intact cells. NMR of whole cells holds promise for helping resolve some unsolved mysteries about how bacteria interact with AMPs. This mini-review will focus on recent studies using ^2^H NMR to study how treatment with AMPs affect membranes in intact bacteria.

## Bridging Biophysical and Functional Studies

Much attention has been given to the mechanisms by which AMPs disrupt the membrane bilayers of bacterial cells, permeabilizing them and dissipating the membrane potential (1–3). However, not all AMPs disrupt membranes via the same mechanism and some AMPs have been shown to have targets other than membranes (4–6). Additionally, there are AMPs that have been shown to modulate the immune response of the host organism (7, 8), in which case they are more properly referred to as host defense peptides (HDPs).

A major challenge in AMP research has been in developing a unified picture of AMP mechanism(s) that is consistent, at least for the particular AMP under scrutiny, with the results from a spectrum of experimental approaches, from simple model systems to whole cells to whole organisms. For example, on the one hand, function is often studied via minimal inhibitory concentration (MIC) assays with bacteria, which indicate the minimum concentration of AMP needed to prevent bacterial growth (9–11). On the other hand, NMR and other biophysical studies provide details of AMP structure and AMP-induced alterations to the bilayer structure, such as bilayer thinning, formation of toroidal pores, solubilizing the membrane into micellar structures, or lipid clustering (1, 12–14). Such “biophysical” studies typically employ model lipid systems with ~1–3 different types of lipids. Likewise, relating an AMP's membrane disruption mechanism in one model lipid system with its behavior in a different model lipid system is not always straightforward. As pointed out by Bechinger and Lohner (3, 15, 16) the lipid structure promoted by a particular AMP is perhaps best thought of in terms of a phase diagram, where the lipid arrangement promoted by the AMP is a function of several parameters including peptide-to-lipid ratio, intrinsic curvature of the lipids, temperature, salt, and pH. This way of thinking has the potential to unify findings when a particular AMP is observed to promote one type of lipid structure under one set of conditions, but a different type of lipid structure under a different set of conditions.

In order to compare AMP study results from cells to those from liposomes, a number of workers have tried to determine, from experimental data, the molar AMP to lipid (AMP:L) ratio needed to see growth inhibition in cells and the AMP:L ratio needed to see liposome disruption *in vitro*. A decade ago, Wimley estimated that for typical experimental conditions the molar bound AMP:L ratio was about 1:200 for liposomes and about 10–100:1 for cells (17). Around the same time, Melo et al. (18) used partition constants to link the two types of experiments. For the two AMPs for which they had both *in vitro* and *in vivo* data, omiganan and melittin, they found that the cell-bound AMP:L ratio was 2.3–9.2 times higher than the threshold needed to see effects on liposomes. As reviewed in (19), the amount of cell*-*bound AMP at the minimum bactericidal concentration (MBC) has been measured via fluorescently labeled AMP or via separation of unbound and cell-bound AMP via centrifugation. Depending on the peptide, the AMP:L ratios for binding to *E. coli* ranged from ~1:3 to 5:1.

**Figure 1:**
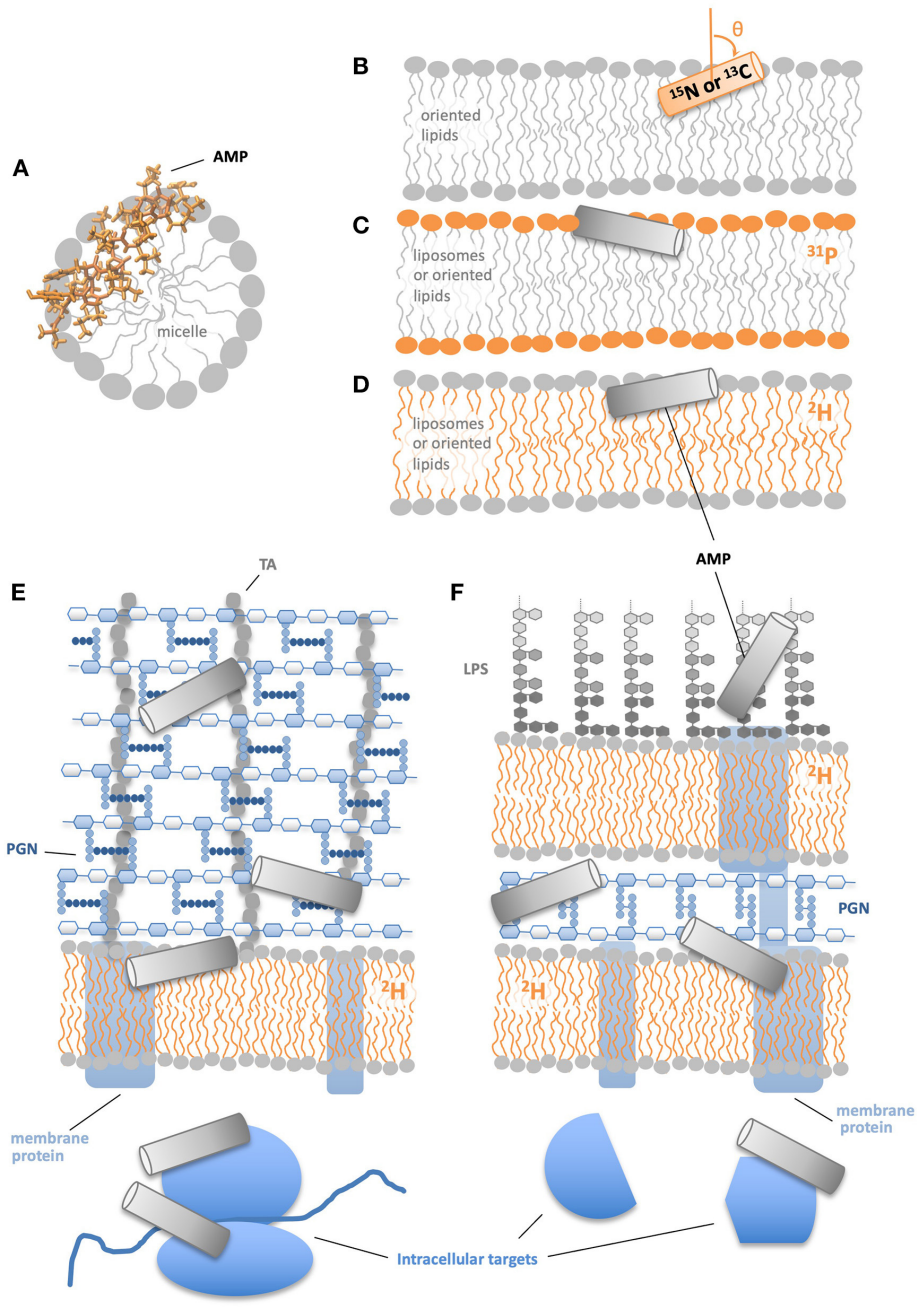
NMR approaches to study AMP structural mechanisms. **(A)** Solution NMR provides atomic resolution structures of AMPs in micelles. **(B)** Solid state NMR of ^15^N or ^13^C labeled peptides in physically oriented bilayers indicates residue-specific helicity and angle of helical segments relative to the bilayer. **(C)** ^13^P solid state NMR of bilayers with a AMPs shows the effect of AMPs on lipid head groups. **(D)** ^2^H solid state NMR of AMPs in bilayers show the effect of AMPs on lipid acyl chains. ^2^H NMR of membrane deuterated Gram(+) **(E)** and Gram(–) bacteria **(F)** indicate how AMPs' effects on lipid acyl chains are modified by non-lipid cell components.

There are a number of potential reasons for a difference in AMP:L ratios between *in vitro* and *in vivo* studies. For instance, some AMPs may bind targets in addition to lipids, including intracellular targets (20–25), and/or non-lipid components of the cell envelope, such as lipopolysaccharide (LPS), peptidoglycan (PGN), teichoic acids (TA), or membrane proteins (Figures 1E,F) (26–30). With regards to cell envelope interactions, two divergent potential effects have been suggested. One possibility is that non-lipid cell envelope components may entrap AMPs, sequestering them away from the lipid bilayer and thus protecting the cell. On the other hand, the non-lipid cell envelope components, especially those with a net negative charge, may attract more AMPs toward cells, leading to more AMP accumulating on the lipid bilayer and thus more damage.

Another aspect of AMP studies where it is vital to link the basic research with model lipid systems that probe AMP mechanism to AMP behavior in more complex systems, is in optimizing AMPs for systemic use in humans. Physiological levels of salt may substantially reduce the membrane-disrupting activities of AMPs (31, 32). AMP binding to serum proteins may reduce their availability to bind the target cells, e.g., bacteria or cancer cells (33, 34). Protease activity may reduce the half-life of peptides in the bloodstream, which, interestingly, could be counteracted by AMP aggregation (34–36). Particularly for histidine-rich peptides, pH can have a large impact on activity (9, 37–39), which can be exploited to confer increased AMP activity around tumors where the pH is low (40, 41) or in helping AMPs escape lysosomes/endosomes (42–44). And of course, optimizing the selectivity of AMPs toward the target cells and minimizing host cell toxicity is always of paramount concern.

In order to understand the fundamentals of how this important class of molecules function, as well as to effectively deploy AMPs in the clinic, it is critical to address the afore-mentioned gaps between the *in vivo* function of AMPs, with detailed studies of AMP mechanism in model lipid systems. This objective is starting to be addressed with a variety of approaches that provided high resolution data on AMPs interacting with whole cells, including atomic force microscopy (45–47), electron microscopy (48, 49), Fourier Transform InfraRed (FTIR) spectroscopy (46), differential scanning calorimetry (DSC) (21), and confocal microscopy with fluorescently labeled peptides (50, 51). However, the rest of this mini-review will focus on ^2^H solid state NMR studies of AMPs interacting with whole cells.

## NMR Approaches for Studying AMPs Interacting With Whole Cells

NMR has a number of advantages for studying AMP mechanisms: (1) it provides atomic resolution data on the structure of both the peptide and lipid components of the system; (2) NMR can be used to characterize the dynamical behavior of peptides and lipids; (3) NMR experiments can be carried out in physiological-like solution conditions; (4) the isotope labels on the lipids and peptides have very little potential to disturb the systems under study, in contrast to, for example, fluorescent labeling; and (5) isotope labels provide the ability to observe selected molecules, i.e., peptides or the lipids, within the context of much more complicated systems, including whole bacteria. For these reasons, there are many NMR studies of AMPs in the literature, although by far the greatest number are in model systems, rather than in whole cells.

There are a variety of ways NMR has traditionally been employed to study AMPs in model systems (Figures 1A–D). Solution NMR can supply atomic resolution structures of AMPs in solution or, more commonly, in membrane-mimetic systems such as detergent or lyso-lipid micelles (Figure 1A) (52–56). Solid state NMR of ^15^N- or ^13^C- labeled AMPs in physically oriented bilayers provides residue-specific information on the helicity of the AMP as well as the angle between the helical segment(s) and the bilayer normal (Figure 1B) (3, 57–60). Complementary to the information provided by NMR-active nuclei within the AMP, solid state NMR in liposome or oriented lipid samples also offers structural and dynamical data on the lipids in the system. ^31^P-NMR is frequently used to report on the behavior of the lipid headgroups (Figure 1C), while ^2^H-NMR with acyl chain deuterated lipids reveals the structure and dynamics at specific locations along the acyl chain (Figure 1D). With ^31^P-NMR one can learn about AMP-induced changes in phospholipid headgroup structure and dynamics, as well as probe for preferential interactions between the AMP and individual components of lipid mixtures (60–62). ^2^H-NMR is commonly used to observe AMP-induced alterations in the order parameter profile of the deuterated lipid acyl chains and in many cases indicates that the presence of the AMP disturbs the acyl chains in a manner consistent with the AMP positioning near the polar/apolar interface (63–65). Solid state REDOR NMR is used to measure the distance between an isotope labeled nucleus on an AMP to specific atoms in the lipids, e.g., ^31^P or ^13^C (66, 67). ^1^H and ^19^F spin diffusion have been used to measure AMP to lipid distances and determine AMP oligomeric state in the bilayer (68, 69).

NMR approaches have been adapted for the study of whole cells in a variety of ways. One relatively well-developed approach is the application of solution NMR to proteins or nucleic acids inside whole cells that range from bacterial to human cells (70, 71). Solution NMR strategies include recombinant expression of the protein of interest, or delivery of the proteins from the outside via electroporation or linkage to cell penetrating peptides (72–74). Solution NMR has also been employed to probe AMP binding to the fungus *C. neoformans* as well as to probe AMP-DNA binding via ^1^H NMR of whole cells (75). Membrane proteins and large, soluble proteins in whole cells and whole organelles have been studied with solid state NMR techniques and have benefited from developments like amino acid selective isotope labeling and sensitivity enhancement from dynamic nuclear polarization (76–79). Magic Angle Spinning (MAS)-NMR has been used to study the carbohydrates in the cell envelopes of both unlabelled and selectively isotope-labeled bacteria, including how the carbohydrates are affected by antimicrobial agents (80–82). The molecular architecture of intact fungal cell walls has been probed via ^13^C correlation spectroscopy (83, 84). ^13^C MAS spectra report on both the PGN and TA components of cell envelopes and ^15^N MAS reveals details of the peptidic components of the cell envelope. Two-dimensional ^13^C NMR has also been used to study starch granules in intact cells (85). Overall et al. (86) have shown how ^31^P can be used to study AMPs' interactions with whole cells. In this context ^31^P reports primarily on nucleic acids, but also contains some information on phospholipid headgroups. The Booth and Marcotte groups independently pioneered ^2^H-NMR methods to study AMPs interacting with membrane-deuterated bacteria (87, 88). The remainder of this mini-review will focus on the ^2^H-NMR work in intact cells.

## ^2^H NMR of Membrane-Deuterated Bacteria

The first ^2^H-NMR spectra of membrane-deuterated bacteria were attained in the early 80s by the Davis group (89). In more recent work aimed at using ^2^H-NMR to study how AMPs interact with bacteria (Figures 1E,F), researchers have employed two different approaches to incorporating ^2^H-labels into the bacterial membranes. The first strategy uses a mutant strain of *Escherichia coli (E. coli)*, unable to either metabolize or synthesize fatty acids (87). The mutant bacteria are grown in the presence of deuterated palmitic acid (PA) and un-deuterated oleic acid. The second approach employs unmutated bacteria [Gram(+) or Gram(–)] which, during growth, are supplied with deuterated PA complexed with dodecylphosphocholine (DPC) micelles to facilitate uptake of the PA (88, 90). For the bacteria to remain healthy and maintain a normal acyl chain composition in their membranes, it is important to also provide oleic acids in the correct proportion to PA, which varies depending on the type of bacteria (90, 91). The two methods of isotope labeling lead to very similar, but not identical spectra of *E. coli*, likely due to variations in lipid composition introduced by the different growth protocols (Kumari, Morrow, and Booth, publication in preparation). Thus far, the approach has been applied to both Gram(–) and Gram(+) bacteria in the absence and presence of AMPs, as well as to microalgae (87, 90–98). The viability of the bacteria during NMR data acquisition depends both on optimization of the growth conditions, as well as the length of the NMR experiment, but, with care, ~80% of the bacteria remain able to metabolize and divide, even after 8 h in the NMR spectrometer at 37°C (87). Moreover, the NMR spectra obtained from the cells remain largely unchanged up to ~10 h after the cells are prepared.

**Figure 2:**
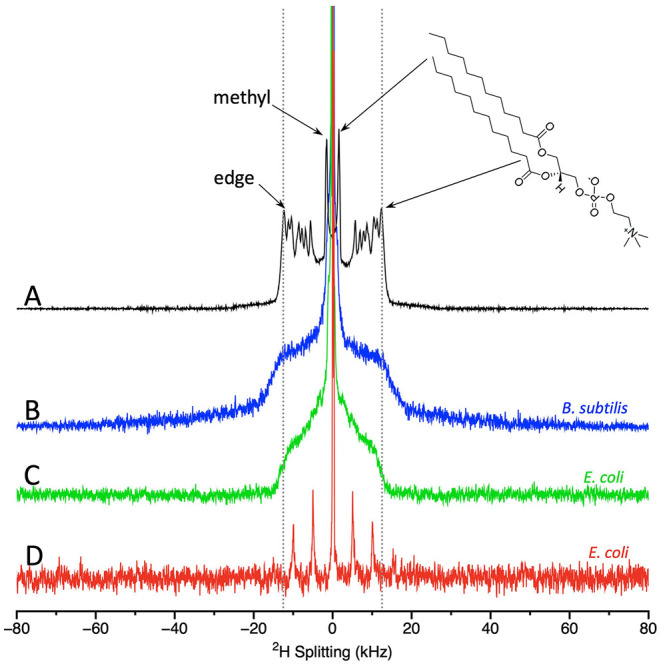
Static solid state NMR spectra of **(A)** model lipids, i.e., dilauroyl phosphatidylcoholine-d_46_ (DLPC-d_46_); **(B)** ^2^H-labeled *B. subtilis;* and **(C)** ^2^H-membrane labeled *E. coli*. MAS NMR spectra of **(D)** ^2^H-membrane labeled *E. coli*. DLPC spectra acquired at 25°C and the bacterial spectra acquired at 37°C. DLPC spectrum provided by Michael Morrow and Tim Porter. *B. subtilis and E. coli* static spectra as in (91, 99) provided by Nury Paula Sanisteban. MAS *E. coli* spectrum provided by Sarika Kumari.

Two types of NMR spectra can be obtained from the membrane-deuterated bacteria, static spectra and MAS spectra. Both types of experiments provide information of key importance to understanding how AMPs interact with membranes. From the NMR spectra, it is possible to derive the degree of acyl chain order and thus the amount of membrane disruption induced by the AMP. Figure 2 shows static spectra for the Gram (–) bacteria *E. coli* and the Gram (+) bacteria *Bacillus subtilis (B. subtilis)*, MAS spectra for *E. coli*, and for comparison, a static spectrum with lipids alone. Starting with the lipid-only spectrum, the key features to note are as follows. There is a prominent edge at ~±12.5 kHz that derives largely from the acyl chain deuterons located near the lipid head groups. The deuterons at the opposite end of the acyl chain, i.e., the methyl groups, give rise to the intense pair of peaks near the center of the spectrum. Offering attention to these two regions of the spectra serves to illustrate the most important feature of ^2^H-NMR of lipids, especially as applied to the study of AMPs; large splittings correspond to greater orientational ordering of the lipid acyl chains with respect to the bilayer normal, while small splittings indicate disorder. Thus, the deuterated methyl groups in the disordered center of the bilayer give rise to small splittings, whereas the deuterons on the acyl chains near the head groups are more ordered and thus contribute to peaks with larger splittings. The essential takeaway for application of the technique to AMPs, is that the bilayer disruptions caused by AMPs are generally observed as a narrowing in the splittings.

Turning to the static spectra of the membrane deuterated bacteria (Figures 2B,C), it is clear that many of the finer details seen in the lipid-only spectra are lost. This outcome is not surprising given that in bacteria the deuterons will be found on different types of phospholipids, and even the same phospholipids may well be located in different microenvironments. However, some key features of the spectra are retained. The prominent edge at ~±12.5 kHz (from deuterons near the headgroup) can still be observed with the same splitting as for the lipid-only samples. The methyl groups can also be observed in the spectra of *E. coli*. Although the spectra from *E. coli* and *B. subtilis* share the prominent edge at ~±12.5 kHz, consistent with lipids in liquid crystalline phase, there are differences in the shape of the bacterial spectra between ~±4–8 kHz. MAS NMR of *E. coli* provide similar information to the static spectra and have the significant advantage of a much shorter acquisition time (98). The MAS spectrum of *E. coli* display a central peak plus 3 pairs of spinning sidebands (Figure 2D). To compare the spectra, especially those from MAS to those acquired statically, it is useful to extract quantitative measures from the spectra.

**Figure 3:**
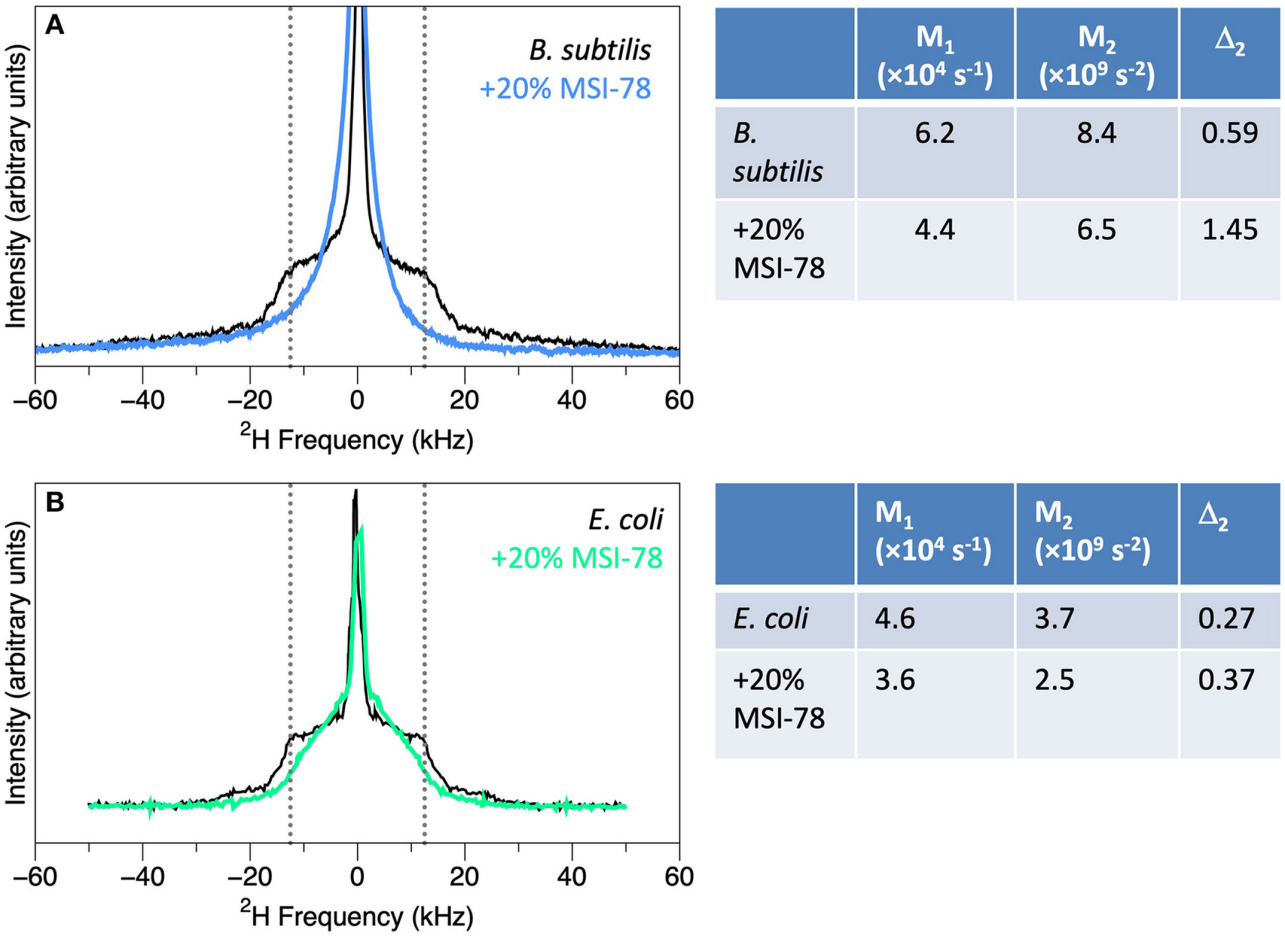
Spectra of membrane-deuterated *B. subtilis* **(A)** and *E. coli* **(B)** without (black) and with the addition of 20% (by weight) of AMP MSI-78 (blue and green), along with the M_1_, M_2_, and Δ_2_ values calculated from the spectra. *B. subtilis* spectra from Nury Paula Santisteban as in (91) and *E. coli* spectra from James Pius as in (87).

The measures in common use are the first and second moments, M_1_ and M_2_, as well as Δ_2_, a parameter derived from M_1_ and M_2_ (right-hand panel of Figure 3). M_1_ and M_2_ are proportional to the frequency-weighted averages of the lipid acyl chain order parameters. Thus, larger values of M_1_ and M_2_ indicate relatively well-ordered lipid acyl chains. Δ_2_ is a useful measure of the overall shape of the spectra (95, 100). As an example, these quantitative parameters provide a way to assess an important issue for living, complex and sensitive biological samples, i.e., how consistent the spectra and moments are from sample to sample. This has been characterized, in particular by Santisteban et al. (91) who found that for 6 sample preparations of *B. subtilis*, the standard deviation in Δ_2_ was 5%, while for 5 sample preparations of *E. coli it* was 9% (99).

## What ^2^H-NMR of Whole Cells Has Taught Us About AMP-Cell Interactions So Far

Addition of AMPs to bacteria leads to striking changes in the ^2^H NMR spectra, indicating substantial disruption of the lipid bilayers. Static spectra of *B. subtilis* and *E. coli* with and without 20% (by dry weight of bacteria) of the AMP MSI-78 are shown in Figure 3. MSI-78 causes a reduction in the intensity at large splittings with a concomitant increase in intensity at the smaller splittings, indicating the peptide induces disorder in the lipid acyl chains. These changes can be quantified as reduction in M_1_ and M_2_, and an increase in Δ_2_ with the addition of AMP (Figure 3). Similar AMP-induced changes are seen in ^2^H spectra of model lipids, e.g., (101). What is remarkable is that the same observation can be made in the context of whole, intact, living bacteria.

In addition to MSI-78 (87, 90–92, 95), ^2^H-NMR of membrane deuterated bacteria has also been performed with the AMPs CAME, BP100 (91, 95), caerin 1.1 and aurein 1.2 (94), as well as antibiotics polymyxin B and fullerenol nanoparticles (88). All the AMPs tested thus far induce similar changes in the NMR spectra, consistent with similar peptide-lipid interactions. One way to consider the uniqueness of AMPs' effects on lipid bilayers in bacteria is to compare their effects to other means of disrupting lipids. Neither mechanical lysis of cells, nor organic solvent lipid extraction leads to alterations in the ^2^H NMR spectra (91, 99). Hence it appears the AMPs' effects on lipids are quite distinctive, and that intact, living cells are unable to repair the damage from AMPs, unlike the self-repair of the bilayer that happens after mechanical lysis or lipid extraction.

Perhaps the most instructive aspect of the work thus far is the consideration of the bound AMP:L ratios needed to see changes in the ^2^H-NMR spectra of intact bacteria. Assuming that most of the MSI-78 binds to the cells, which is reasonable given the large positive charge of the peptide, the high concentration of cells during treatment, and low amount of protein measured in the supernatant after the AMP-treated cells are centrifuged, about thirty times more peptide is required to see lipid disruption in intact cells than is needed in ^2^H-NMR studies of AMPs in model lipid systems (87, 101). Consequently, there must be something present in the cells that is protecting the bilayer from disruption, either by directly stabilizing the bilayer, and/or by sequestering AMPs away from the bilayer. And whichever cell component(s) this effect is coming from, it seems to be present in both Gram(+) and Gram(–) cells. Possibilities abound (Figures 1E,F). Non-lipid components of the cell envelope such as LPS, PGN, or TA could be stabilizing the bilayer and/or sequestering the AMPs away from the bilayer. Membrane proteins and intracellular molecules are also potential targets for AMPs. In fact, MSI-78 has been shown to disrupt the thermal stability of ribosomes and inhibit transcription (21). Thus, the work with the limited selection of AMPs proved via ^2^H-labeled whole cell NMR so far is consistent with a multi-hit mechanism (1, 5, 17, 20, 102–104). Conversely, there are several AMPs for which the biological activity of the L- and D-amino acid versions of the peptide are similar [reviewed in Savini et al. (19)], arguing that if these peptides have additional targets beyond the membrane, the interactions are not specific enough to be disrupted by the switch to the alternate enantiomer.

Turning next to the other end of the AMP:L ratio spectrum, for MSI-78 the AMP:L ratio needed to see membrane disruption in ^2^H-NMR spectra (~1:1) of intact cells is of the same order, but slightly greater, than the predicted values of cell-bound AMP:L (1:2.5–28:1) for a suite of 6 AMPs (18) and the observed membrane-bound PMAP-23 at the MBC in cells (19, 105). We have used flow cytometry to analyze cells treated with MSI-78 under conditions identical to the NMR experiments and found that for the AMP:L concentration shown in Figure 3, there is no MSI-78-induced increase in cell permeability. Since the NMR experiments reveal major disruptions to the lipid bilayer at AMP:lipid ratios lower than what is lethal, it seems possible that AMP is getting across the bilayer to the inside of the cells (Figures 1E,F). Again, this is consistent with the suggestion that at least some AMPs have intra-cellular targets, and that for at least some AMPs, membrane disruption may not the only mechanism by which the AMP harms cells.

## Future Prospects

^2^H-NMR of membrane-deuterated bacteria could be expanded in a variety of potentially fruitful ways. Firstly, given that different AMPs are likely to function via different mechanisms or sets of mechanisms, it is important not to over-generalize the results from the limited number of AMPs probed so far. Performing similar experiments with a greater variety of AMPs may help reveal variations in lipid interactions with whole cells. Similarly, it will be interesting to expand the work from AMPs to cell penetrating peptides (CPPs) which transverse the bilayer, but do not induce the membrane permeabilization characteristic of many AMPs (22, 106–108).

The ^2^H-NMR approach can also be adapted to probe the role of non-lipid cell components in modulating AMP-lipid interactions. Preliminary work in our group has been done to manipulate LPS and PGN layers to monitor how disrupting these components affects the cytoplasmic membrane in the absence and presence of AMPs. Gentle disruption of the carbohydrate portion of LPS in Gram(–) bacteria results in a slight increase in lipid bilayer disorder, and slightly sensitizes cells to lipid membrane disruption by AMPs. Similarly, disruption of the PGN component of Gram(+) bacteria causes a slight increase in membrane disorder, but unlike LPS disruption, has no detectable effect on AMP-lipid interactions. Since Gram(+) bacterial cell envelopes also have negatively charged TA, it will be interesting to see how disrupting TA affects interactions with positively charged AMPs.

Another exciting prospect is to broaden the approach from bacteria to eukaryotic cells. Such experiments will need to be optimized to incorporate sufficient levels of deuteration into eukaryotic cell membranes. Given the much larger size of most eukaryotic cells compared to bacteria, and the consequent decrease in the ratio of amount of cytoplasmic membrane to the rest of the biomolecules in the cells, signal-to-noise in the NMR spectra may prove to be a challenge. Focussing on smaller types of eukaryotic cells, or organelles such as mitochondria, may be a more achievable. Another feasible prospect would be to carry out experiments with AMPs and deuterated bacteria in the presence of unlabelled eukaryotic cells, which would give a sense of the selectivity of the AMP for the bacterial membranes. Furthermore, studying AMP-resistant cells with NMR may help reveal how the cell envelope alterations of the resistant cells affect the ability of the AMP to disrupt the lipid membranes.

## Author Contributions

The author confirms being the sole contributor of this work and has approved it for publication.

## Conflict of Interest

The author declares that the research was conducted in the absence of any commercial or financial relationships that could be construed as a potential conflict of interest.
